# Integrated information in the EEG of preterm infants increases with family nurture intervention, age, and conscious state

**DOI:** 10.1371/journal.pone.0206237

**Published:** 2018-10-24

**Authors:** Joseph R. Isler, Raymond I. Stark, Philip G. Grieve, Martha G. Welch, Michael M. Myers

**Affiliations:** 1 Division of Neonatology/ Department of Pediatrics, Columbia University, New York, New York, United States of America; 2 Nurture Science Program, Department of Pediatrics, Columbia University, New York, New York, United States of America; 3 Developmental Neuroscience, Department of Psychiatry, Columbia University, New York, New York, United States of America; TNO, NETHERLANDS

## Abstract

A putative quantifier of consciousness, integrated information, was applied to preterm infant EEG data after novel pre-processing. Integrated information had a non-random structure as a function of the time lag over which it was computed. For most lags, it increased with age in early life, but even more so in infants exposed to Family Nurture Intervention (FNI), providing further evidence that FNI advances brain maturation in preterm infants. Also, it discriminated between conscious states (awake, REM sleep, NREM sleep), providing empirical support for the Integrated Information Theory of Consciousness in human infants.

## Introduction

Determination of the degree of consciousness in the clinical setting presents challenges for individuals who cannot perform tasks or make verbal reports, such as those in coma, under anesthesia or infants early in development. Qualitative scales of the degree of consciousness have been developed for comatose patients [1, 2], and a proprietary EEG monitor is used in some operating rooms for measuring depth of anesthesia [3]. However, other than assessments of awake versus sleep, we know of no studies that quantify the degree of consciousness in early infancy. In this report a recently proposed measure of integrated information in time series data from the electroencephalogram (EEG) is used to evaluate levels of consciousness in prematurely born infants during hospitalization in a Neonatal Intensive Care Unit (NICU).

The integrated information theory of consciousness (IITC) is an evolving approach to quantifying consciousness being developed by Giulio Tononi and colleagues [4–7]. Some theories of consciousness view it as an epiphenomenon of brain activity with no causal efficacy of its own, i.e. incapable of “top-down” causality [8]. In contrast, a hallmark of IITC is a proposed mechanism by which top-down causality is conceivable [9]. IITC is based on a measure of integrated information, denoted as Phi (Φ). This measure is similar to other proposed measures of consciousness [10–13] that are based on the idea that a brain (network) state underlying consciousness should be simultaneously both highly differentiated locally and highly integrated globally. In IITC, Φ is defined for discrete time-dependent Markovian processes and quantifies the amount of holistic information in a network of interacting elements that is causal to state transitions within the network [7]. Validation of IITC has for the most part come from computational models of network dynamics.

Barrett and Seth [14] pointed out some methodological limitations and conceptual difficulties in applying the Φ of IITC to biological systems. As a result, Barrett and Seth subsequently adopted alternative formulations of Φ to compute integrated information from time series data, such as that contained in recordings of physiological signals. In contrast to the discrete Markovian processes of IITC, they define two formulations of Φ for continuous time processes. Both of these formulations assume time series data with stationary statistics. One formulation (“Φ empirical”) further assumes Gaussian dynamics; however, for this current study we have used their other formulation which does not assume a normally distributed process (“Φ autoregressive”).

The calculation of Φ autoregressive (hereafter Φ) is dependent on a choice of temporal scale tau (denoted τ), namely, the time between two particular states of a network. Conceptually, Φ quantifies the degree to which holistic information in a network of interacting elements predicts a future network state given an earlier state separated in time by a lag (τ). In modeling studies, τ is often chosen to be a single time-step in the computational model. When applying this calculation to continuously sampled physiological time series data, the question becomes: what is the relevant temporal scale of the lag, i.e. what is the most appropriate τ? In adults, there is some evidence that the content of consciousness requires some hundreds of milliseconds (ms) of unconscious processing [15], suggesting a “conscious moment” duration of ~ 50 to 500 ms [16]; but see [17] for an alternative view). However, nothing is known about the duration of a conscious moment early in infancy. Accordingly, we adopted an exploratory approach and computed integrated information over a wide range of lags (τ).

After the analyses in this report were performed, very recent theoretical work has appeared highlighting some inherent difficulties Barrett and Seth’s Φ [18]. In particular, its computational cost can be prohibitive and negative values may occur. Nonetheless, as shown in this report, use of Barrett and Seth’s Φ is both computationally feasible and negative values lead to suggestive results.

Φ was computed from electroencephalographic (EEG) recordings of prematurely born infants in a study of Family Nurture Intervention (FNI) conducted by the Nurture Science Program at Columbia University Medical Center. This randomized controlled trial evaluated a new NICU-based behavioral intervention designed to facilitate maternal/infant emotional connection and physiological co-regulation [19]. Comparisons of mothers and babies who received FNI versus standard care (SC) have shown multiple improvements in later neurobehavioral outcomes [20, 21], including evidence of accelerated maturation of brain activity [22–24]. If indeed integrated information is a measure of consciousness, as proposed in IITC, we posited the following three hypotheses: 1) Φ would increase with age in the perinatal period; 2) given prior findings, FNI would accelerate Φ maturation in infants who experienced the intervention compared to those who did not; and 3) Φ would be higher when waking than sleeping and higher in active sleep than in quiet sleep (infant analogues of adult REM and Non-REM sleep).

## Materials and methods

### Subjects and intervention procedures

The FNI randomized controlled trial was a longitudinal, parallel-group trial in the level IV NICU at the Morgan Stanley Children’s Hospital of New York comparing infants receiving standard NICU care with FNI (ClinicalTrials.gov, NCT01439269). The CUMC Institutional Review Board approved all recruitment, consent, and study procedures and all parents granted written, informed consent to participate in this trial. A complete description of the FNI protocol was published previously [19]. Briefly, 115 families with 150 preterm infants born at 26 to 34 weeks postmenstrual age (PMA) were enrolled over a 42 month period from January 2009 through July 2012. (Infants with the same mother (twins) were considered as independent for statistical analyses.) Mothers who did not speak English, or who had a history of drug addiction or severe mental illness were excluded from the study as were infants with birth weights below the third percentile for gestational age or who had a significant congenital defect. The enrollment, consort chart, and reasons for loss to follow-up for this study can be found in a prior publication [25].

After enrollment and consent, infants were randomly assigned to either the standard care (SC) or FNI groups. Mothers of infants assigned to the intervention group met with Nurture Specialists, former NICU nurses trained in implementing FNI, who guided the mothers and families through repeated calming sessions with the aim of establishing co-regulation and emotional connection. Mothers and infants assigned to SC received the usual care of the CUMC NICU with the frequency and content (e.g. skin-to-skin care, breast milk feeding, psychological and social work support) determined by the mother’s preferences.

The initial FNI mother-infant calming sessions took place when infants were in an incubator. As soon as possible after birth (average of 7 days from birth to first intervention [20]), the mothers were engaged in reciprocal scent cloth exchanges. Two small cotton cloths were given to the mothers, one to wear in their bras to then give to their infants the next day, and the other to place under the head of their infants. Mothers were encouraged to exchange these cloths at each visit to the NICU. The Nurture Specialists then facilitated FNI mothers in making contact with their infants through the ports of the incubator, using firm and sustained touch and tender manual containment, speaking emotionally to their infants in their primary language and, when possible, making eye contact. Later during the NICU stay, but as soon as possible, these activities were continued during skin-to-skin, or non-skin-to-skin holding when the mother preferred. Fathers and grandparents were also encouraged to do skin-to-skin holding. When family members were available, the Nurture Specialists engaged them in sessions that also discussed the importance of the FNI activities between mother and infant, and offered support to the families with the ongoing stresses of a family with a preterm infant in the NICU. Mothers were encouraged to engage in such activities with their babies for at least one hour each session. On average the FNI mothers had 3.5 sessions per week and engaged in these activities ~6 hours/week [25]. In this current study, we report EEG results for 272 60-minute studies (49 SC and 56 FNI infants). Parental demographics and maternal and infant clinical conditions did not differ between these groups.

### State coding

Trained research assistants performed visual coding of behavioral states (hereafter conscious states) during recording of the EEG. State was coded for each minute using behavioral criteria previously shown to be appropriate for preterm infants [26]. States were scored as active sleep (i.e. REM, AS), quiet sleep (non-REM, QS), awake (W), indeterminate, or crying. QS in infants was characterized by regular breathing, no eye movements and the absence of gross body movements. In contrast, AS was characterized by eye movements, irregular respiration, and frequent body twitches. Of 272 studies, AS, QS, and W states were detected in 247, 230, and 34 studies, respectively.

### EEG rRecording and pre-processing

EEG was recorded with a multi-electrode geodesic net (124 leads referenced to vertex) and the NetStation data acquisition system (EGI Inc., Eugene, Oregon). EEG recordings were obtained between 11 am and 4 pm about 30 minutes after a normally scheduled feeding and lasted about 1 hour. During recording, the EEG voltage from each lead (vertex reference) was band-pass filtered in hardware from 0.1 to 400 Hz and digitized with 16 bits per sample at a rate of 1000 samples per second. After recording, data were notch-filtered with a finite impulse response (FIR) filter of order 16,000 for 4Hz wide bands centered on 60 Hz and its harmonics up to 480 Hz and then down-sampled to 250 Hz after applying a FIR low pass filter of order 1000 (down-sampling allowed comparison with other datasets).

To determine the leads and times contaminated by movement-related or other sources of non-cortical electrical artifact, we applied multiple criteria on a second by second basis within each minute. For frequency domain criteria, one second data were demeaned, a Hanning window was applied, and the fast Fourier transform was taken. Criteria were as follows: standard deviation of voltage less than 40 μV and greater than 0.001 μV; sample-to-sample change less than 40 μV; absolute value of voltage less than 100 μV; log-log spectral slope between 20 and 200 Hz less than -0.1 (to screen for muscle artifact). If more than 20 leads had artifact during a second that second was excluded. Remaining data were re-referenced to the average of all leads.

### Calculation of integrated information

A limitation in applying Φ to EEG data is that its computation requires lengthy data segments (ideally thousands of samples) which are needed to construct empirical probability distributions. Thus, Φ computation requires long segments of artifact-free EEG data across a set of data channels. Here, a pre-screening and data reduction method was developed to make the computation of Φ practical for evaluating research and clinical EEG data. An algorithm was developed to find within each minute of data a subset of 12 leads that were simultaneously artifact-free for at least 40 seconds. To protect against any spatial bias, we further required that the subset included one lead from each of 12 spatially disparate scalp areas (see Fig 1 in Myers 2015 [22]). For each minute, channels were sorted based on the number of artifact-free seconds and that sorting was used to determine if the minute contained at least 40 seconds of simultaneously good leads from the 12 scalp areas. If it did, the lead with the best data rate in each area was used; if it did not, the minute was not used. Φ was then computed for each accepted minute, aligned with behavioral state codes, and then averaged within states for each baby. Finally, Φ was computed for 21 values of τ, skewed toward smaller τ and ranging from 1 to 1,500 samples (corresponding to 4 to 6,000 ms).

### Statistical analysis of results

For each value of τ, FNI versus SC group differences were assessed using two sample t-tests and within-group differences with regard to state and τ were assessed with paired t-tests. In this exploratory study, the object was to see if there were non-random structures in the dependence of effects on τ, so all results with p-values less than 0.05 are presented. However, when summarizing the study’s findings, results that remained significant after Bonferroni correction for multiple comparisons (p < 0.0024) are highlighted.

For correlations (Pearson’s linear correlation coefficient) between Φ and postmenstrual age, it was necessary to ensure that only one Φ in each subject is used, i.e. Φ for one value of τ (subjects had from 1 to 5 studies over their NICU stay and so had up to 5 values of Φ for each value of τ). Thus, for every subject with multiple studies we randomly chose one study, performed 1000 permutations, and took the mean correlation across the set of permutations.

All statistical analyses were performed using native Matlab functions. Data pre-processing was performed with custom Matlab programs. The computation of Φ was performed using the Matlab toolkit provided in Barrett and Seth 2011.

## Results

### Relationship of Φ and τ

**Fig 1:**
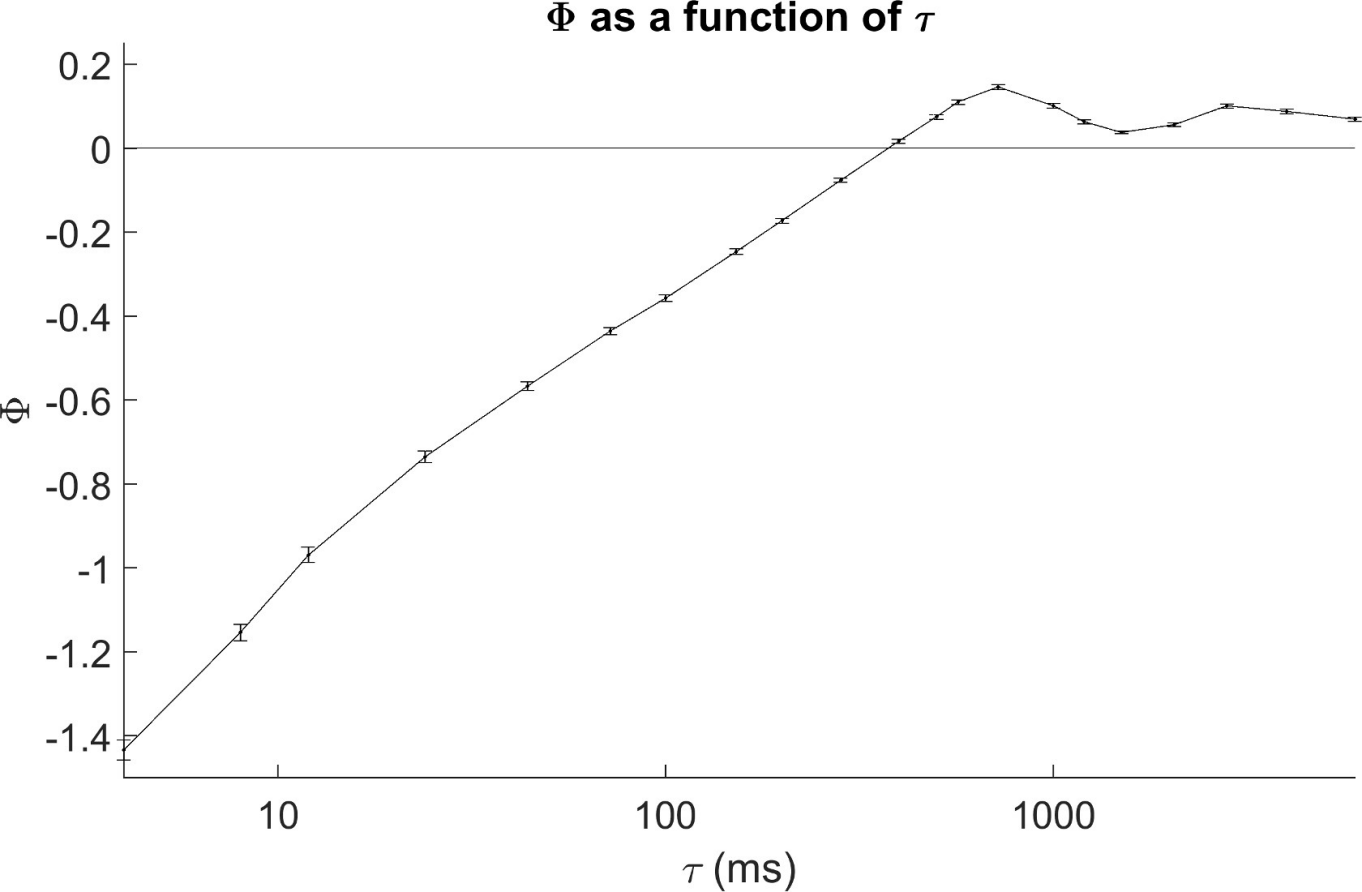
Φ as a function of τ. Mean and standard error of Φ as a function of temporal scale τ. Results were pooled across groups (FNI and SC) and sleep states (AS and QS).

There was clear structure in the dependence of Φ on τ. To illustrate this, results are pooled across all ages and both groups of infants (SC and FNI) and displayed in Fig 1. Mean values of Φ were negative for τ below ~400 ms and positive above. Φ increased linearly with log τ until it became positive, after which it fluctuated between 0 and 0.13 with two broad maxima of 0.13 and 0.1 at τ of ~720 and 2800 ms, respectively.

Of all tests comparing groups, states, and dependence on age, the results with the highest significance (lowest p values) were for the correlation of Φ with postmenstrual age. Accordingly, results for the developmental hypothesis are reported first, then results for the group difference hypotheses, and finally results for the state-dependence hypotheses. In each case, results for two particular values of τ (100 and 1000 ms) are shown and then effects are presented across the entire range of τ.

**Fig 2:**
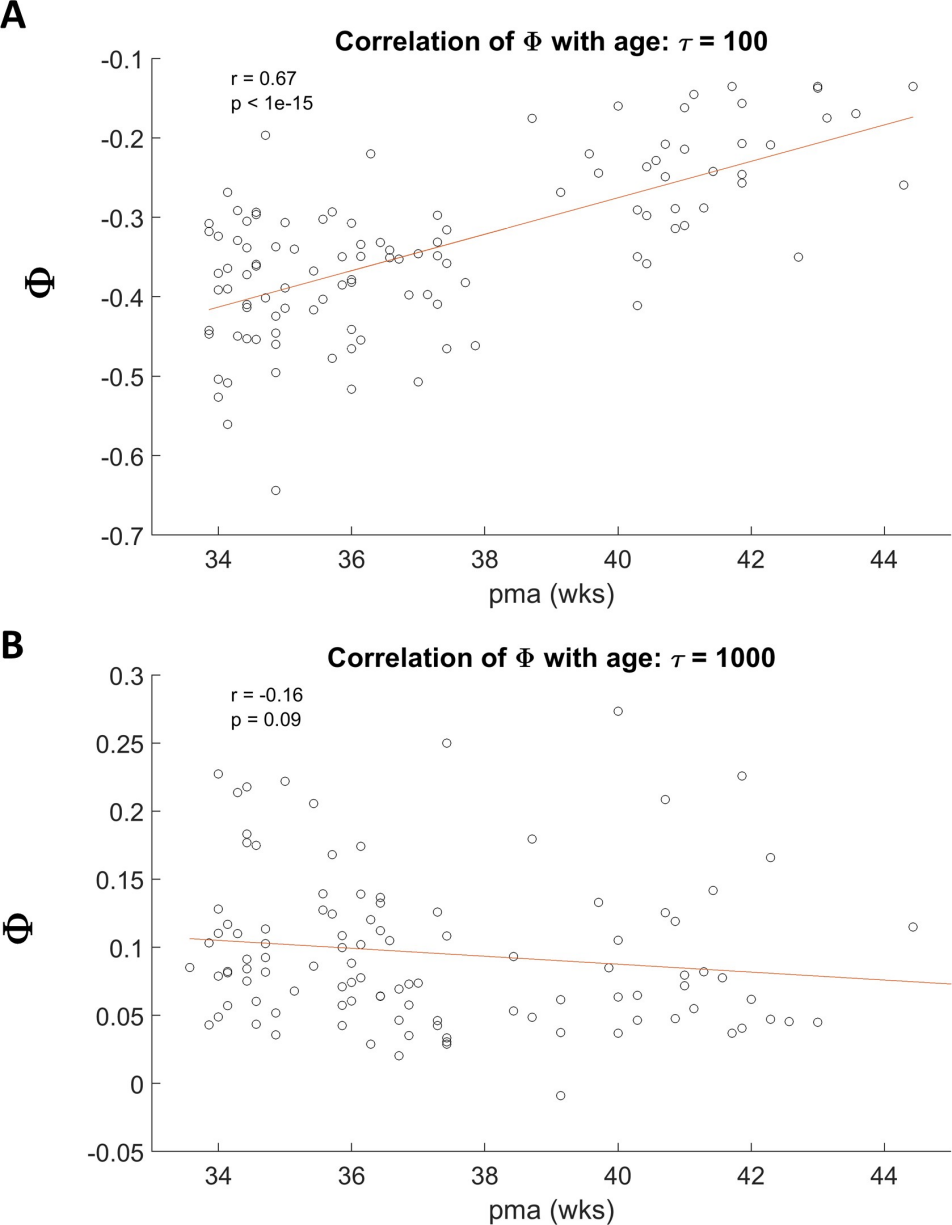
Correlation of Φ with age. Two examples of mean correlations of Φ with postmenstrual age (pma), one for τ equal to 100 ms (**A**) and the other for τ equal to 1000 ms (**B**). In **A**, the correlation coefficient was 0.67 (p < 10^−14^) while in **B** no correlation was found. Results were pooled across groups (FNI and SC) and sleep states (AS and QS).

**Fig 3:**
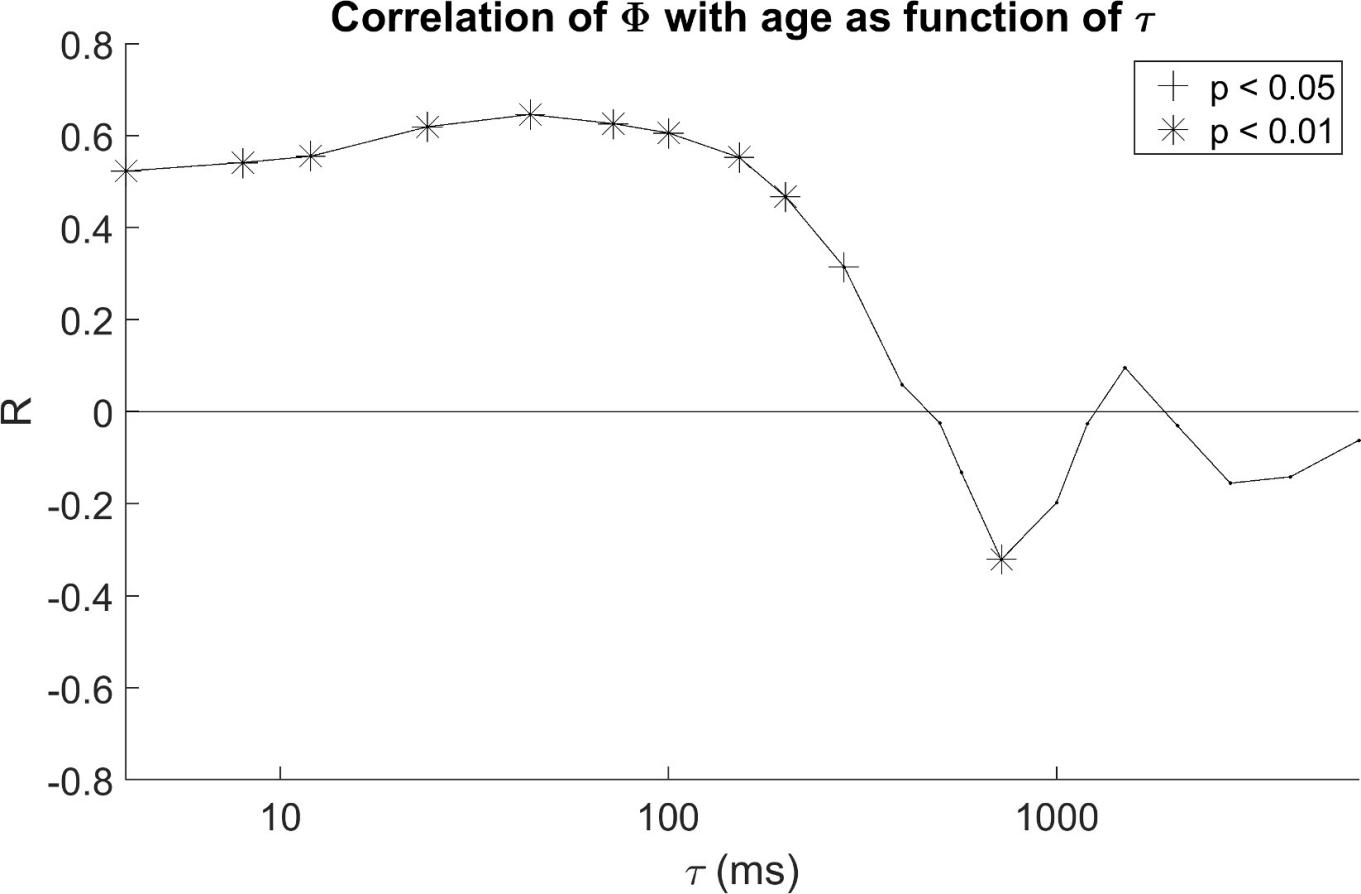
Correlation of Φ with age as a function of τ. Mean correlation coefficients of Φ with postmenstrual age for all computed values of τ. Results were pooled across groups (FNI and SC) and sleep states (AS and QS). Significant correlations are denoted by asterisks (p < 0.01) and crosses (p <0.05).

Fig 2 shows two examples of scatter plots of Φ during sleep (pooled active and quiet sleep) and postmenstrual age (PMA) for the selected values of τ, with superimposed regression lines. In both cases, one of the 1000 permutations using only one study per subject is shown. Fig 2A is for τ of 100 ms and Fig 2B is for τ of 1000 ms. The correlation coefficient at 100 ms is 0.67 (n = 95) and is highly significant (p <10^−15^). In contrast, the correlation coefficient at 1000 ms is slightly negative (- 0.016) and is not significant. Fig 3 summarizes the correlation of Φ with age as a function of τ. Each point in Fig 3 is the mean correlation coefficient of Φ with age over 1000 permutations using only one study per subject for a particular value of τ, with the significance of the correlation shown by its symbol. As hypothesized, Φ increased with age, but only for specific ranges of τ. For a wide range of τ from 4 to ~ 300 ms, Φ was positively correlated with age. The maximum mean correlation coefficient was 0.64 at τ = 44 ms (mean p < 10^−9^). For τ above ~ 400 ms, developmental correlations rapidly transitioned from positive to negative, reaching significance at τ = 720 ms. For a broad range of τ greater than τ 1000 ms, correlations once again tended to be positive.

### Impact of FNI on Φ and τ

**Fig 4:**
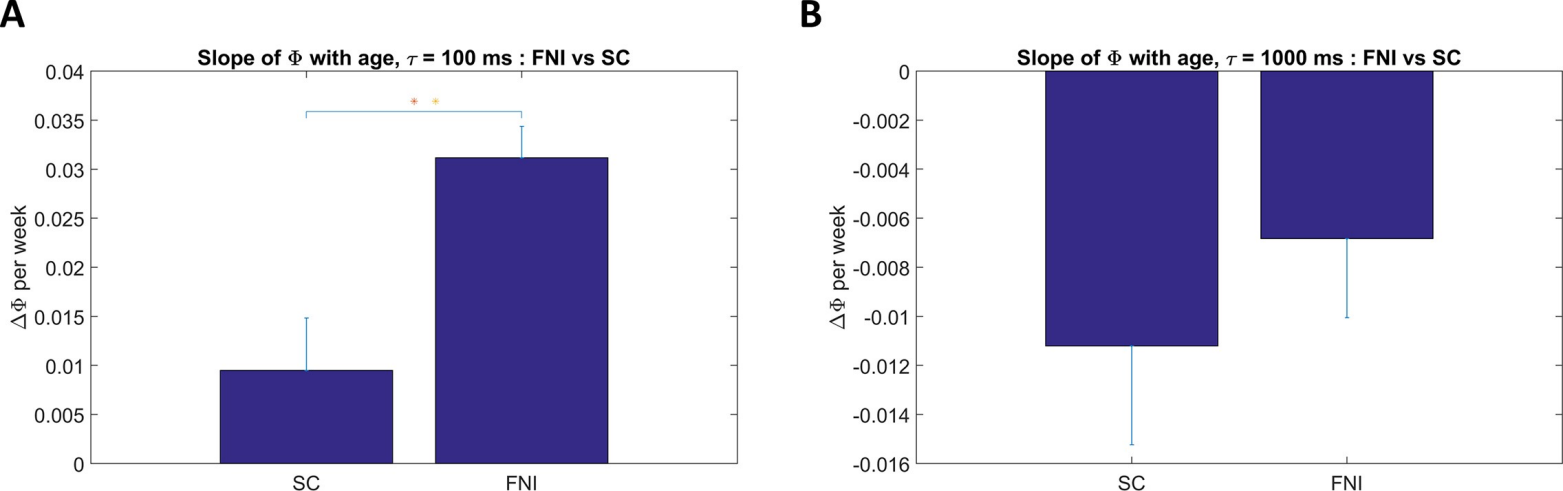
Slope of Φ during sleep. Two examples of group comparisons (FNI vs SC) using best-fit slopes of Φ with postmenstrual age, for the same two values of τ used in Fig 2. Results were pooled across sleep states (AS and QS). Significant differences (two sample t-tests) are denoted by single (p < 0.05) or double asterisks (p < 0.01). **A**: τ = 100 ms, **B**: τ = 1000 ms.

**Fig 5:**
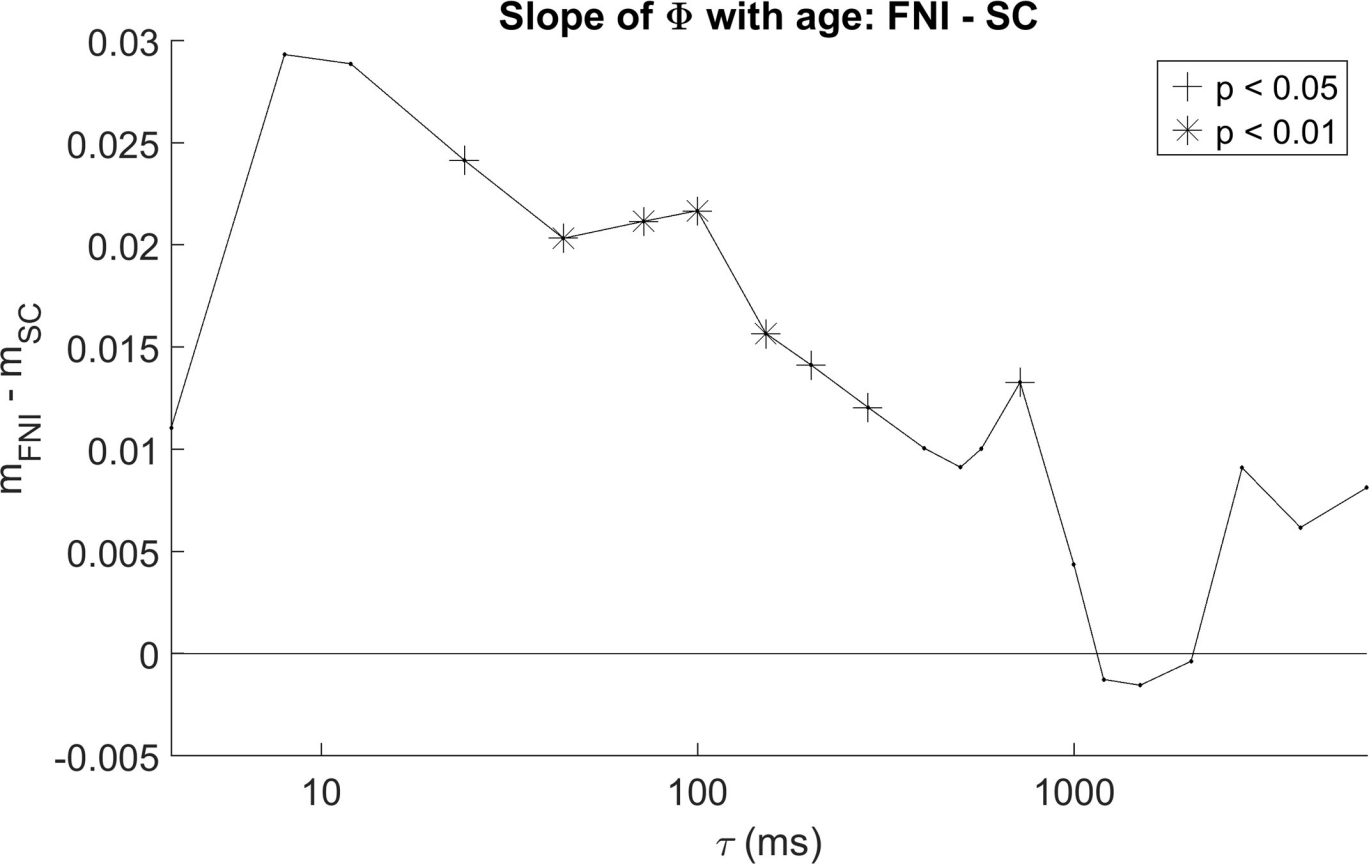
Group differences in Φ by age during sleep. Group comparisons (FNI minus SC) of slopes of Φ with postmenstrual age for all computed values of τ. Results were pooled across sleep states (AS and QS). Significant differences (two sample t-tests) are denoted by asterisks (p < 0.01) and crosses (p < 0.05).

To test the FNI versus SC group difference hypotheses, for each baby studied at more than one age we computed the slope of best fit for Φ during sleep (active and quiet sleep pooled) versus PMA. Fig 4 shows bar plots of slope of Φ during sleep with age compared between groups, for the same two τ’s as in Fig 2. For τ of 100 ms (4A), the mean slope for the intervention group was ~0.03 per week compared to 0.01 per week for the standard care group, with a significant group difference (p<0.001). For τ of 1000 ms (4B), mean slopes were negative in both groups but the group difference was not significant. Fig 5 summarizes group differences in slope of Φ by age during sleep across the full range of τ. Each point in Fig 5 is the group mean difference (mean slope of Φ in FNI group minus mean slope of Φ in SC group), with significance shown by its symbol. For most values of τ between 24 and 1000 ms, the slope in the FNI group was significantly greater than that in the SC group. The maximum group difference was ~ 0.025 (p<0.02) at τ of 24 ms. At and above τ = 1000 ms, where the correlations of Φ with age were weaker (Fig 3), group differences are not significant.

**Fig 6:**
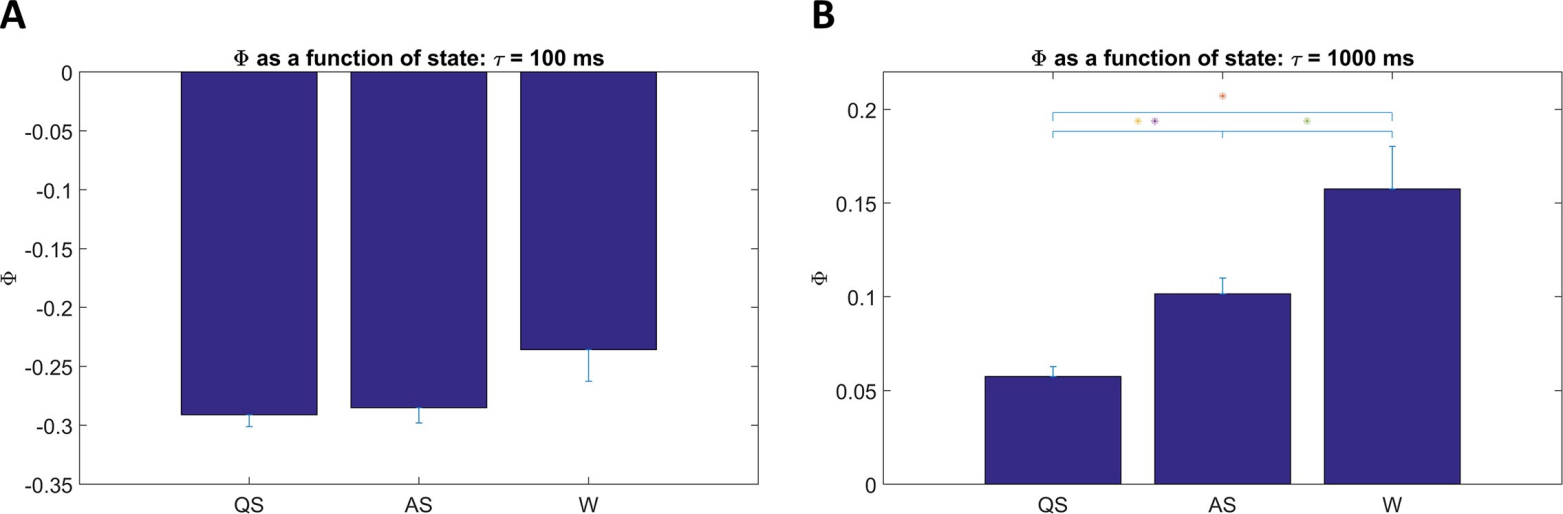
Φ as a function of state. Two examples of behavioral state comparisons (QS vs AS vs waking), for the same two values of τ used in Fig 2. Results were pooled across groups (FNI and SC). The median over studies from 37 to 44 weeks postmenstrual age was used for each subject. Significant differences (paired t-tests) are denoted by single (p < 0.05) or double (p < 0.01) asterisks. **A**: τ = 100 ms, **B**: τ = 1000 ms.

**Fig 7:**
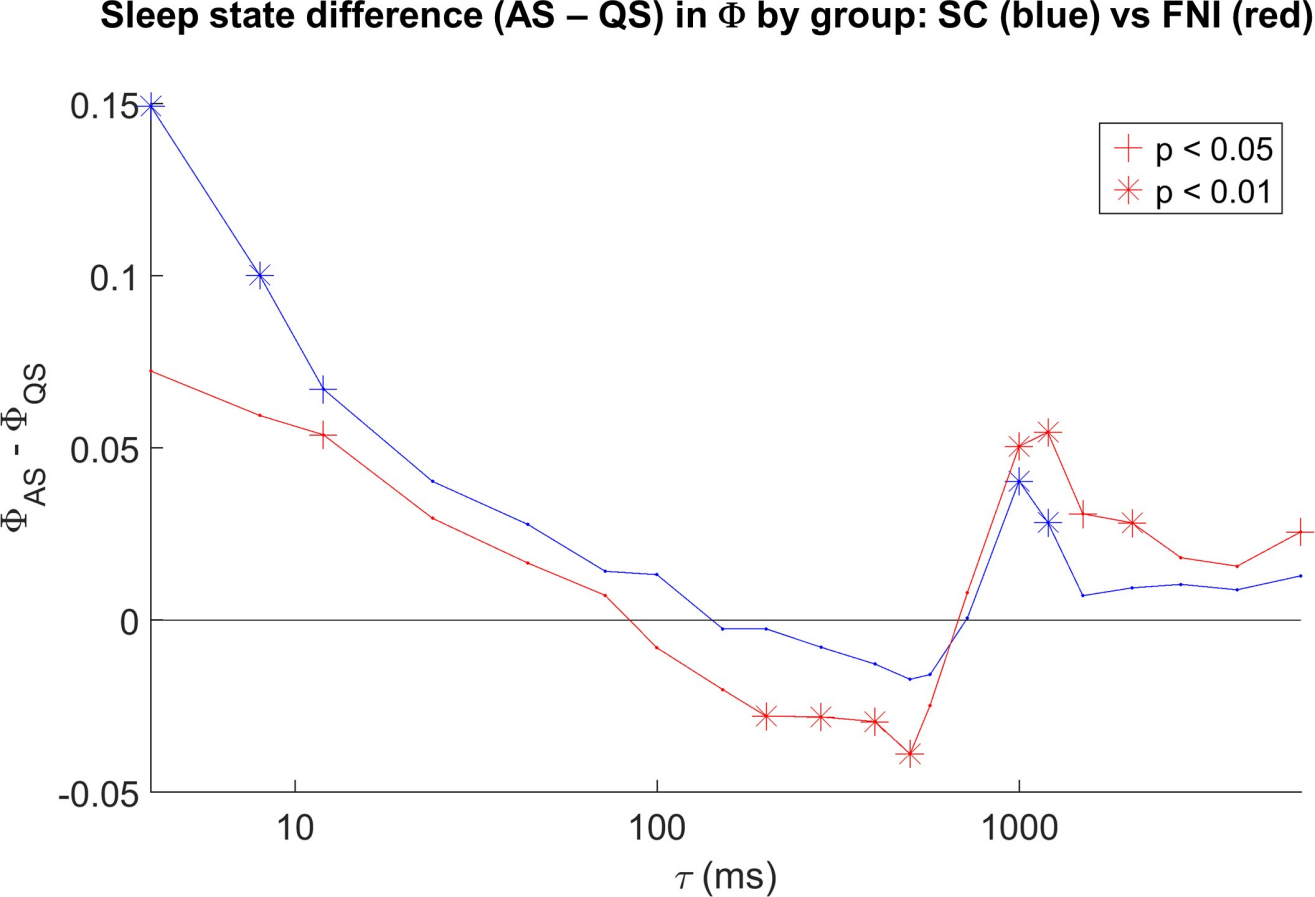
Sleep state differences for all computed values of τ. Results for the FNI and SC groups are shown separately in red and blue, respectively. The median over studies from 37 to 44 weeks postmenstrual age was used for each subject. Significant state differences (paired t-tests) are denoted by asterisks (p < 0.01) and crosses (p < 0.05).

**Fig 8:**
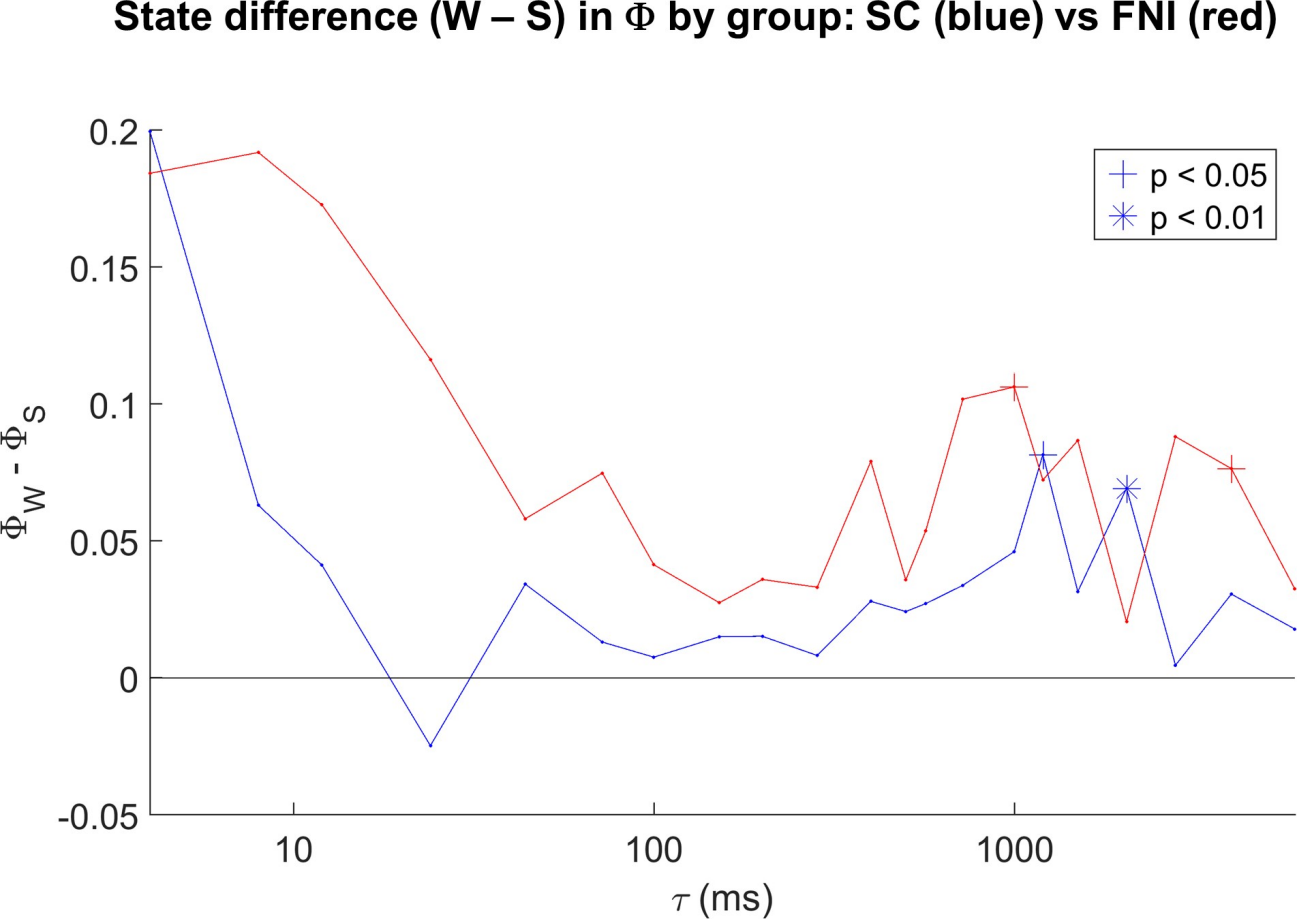
Sleep/wake differences for all computed values of τ. Results for the FNI and SC groups are shown separately in red and blue, respectively. The median over studies from 37 to 44 weeks postmenstrual age was used for each subject. Significant state differences (paired t-tests) are denoted by asterisks (p < 0.01) and crosses (p < 0.05).

To test whether Φ differs between sleep/wake states when the infants were near to term age (37 to 44 wks PMA), the median Φ for each state (QS, AS, W) was computed within each baby across studies within that range, and intervention and standard care group data were pooled. Fig 6 shows bar plots of the resulting state differences (paired t-tests for each pair of states), for the same two τ’s in Fig 2 (100 and 1000ms). For τ of 100 ms (6A), mean Φ values were negative in all three states (see Fig 1), with no significant differences between states. In contrast, for τ of 1000 ms, mean Φ values during W, AS, and QS were ~0.16, 0.1, and 0.06, respectively, with all differences between pairs of states significant. Fig 7 summarizes the sleep state dependence of Φ across the full range of τ, controlling for group. Each point in Fig 7 is the within-group sleep state difference (Φ in AS minus Φ in QS), with significance shown by its symbol. Results for the FNI group are shown in red and the SC group in blue. State dependence tended to be as hypothesized at lower values of τ (i.e. Φ greater in AS) and also in a wide range of τ above ~800 ms in both groups, reaching significance more often in the FNI group. Interestingly, state dependence tends toward the opposite relationship in both groups in a range of τ from ~ 200 to 500 ms (i.e. Φ in QS > Φ in AS), reaching significance in the FNI group. Fig 8 is in the same format as Fig 7, but for wake versus sleep (Φ in wake minus Φ in sleep). Wake and sleep comparisons were always as hypothesized when significant. Comparing Figs 7 and 8, note that there was a much lower number of subjects with at least 3 minutes of waking (9 in the SC group and 6 in the FNI group) than the number of subjects with at least 3 minutes of both/either sleep states (> 40). In general, regardless of which states were compared, the FNI group was more likely to show significant state differences in Φ.

## Conclusion

The question of when in early life consciousness emerges and what factors affect this timing is unknown but is of fundamental interest in the field of developmental neuroscience.[27] It has been proposed that causal holistic information in a brain network (Φ) indexes the level of consciousness of an organism.[4–7] This proposal is supported by adult human research showing correlations between Φ and depth of anesthesia [28], emergence from coma [29], and sleep state [30]. Here we explore the usefulness of measuring Φ in the EEG of very young infants to provide new knowledge about these issues.

To our knowledge, our report is the first description of the relationship between Φ and τ over a wide range of τ in neurobiological data. The computed values of Φ in these preterm infants were found to have a high degree of structure as a function of τ with values of Φ ranging from ~-0.14 to ~+0.13. The range of positive values of Φ obtained from our 12 node (lead) EEG network for lags (τ’s) greater than ~ 400 ms were between 0 and 0.13 (see Fig 1). This range is similar to the values of Φ in the 10 node model networks simulated in Barrett and Seth [14] for τ equal to 1 *dimensionless* time-step. For negative Φ there was a roughly linear dependence log τ, while for positive Φ there were two maxima at ~ 720 and 2800 ms. The demonstration of positive values of Φ suggests there is a causal holistic contribution to total information in the EEG network for τ above ~ 400 ms. Negative values for Φ were seen at shorter (< 400 ms) values of τ. This would suggest that at short τ holistic causal information is less that would be expected based on the sum the parts, i.e. *the sum of the causal information in the individual nodes and all sub-networks is greater than that in the network as a whole*.

This study focused on testing three hypotheses of how variation in τ would affect results: 1) Φ in the infant EEG network increases with age; 2) Φ increases more rapidly with age in infants given greater amounts of maternal nurture while in the NICU; and 3) Φ is greater when awake than during sleep, and greater in AS than in QS (non-REM) sleep. Each of these hypotheses was supported by results that were dependent on τ.

With regard to the effects of age, results showed that at τ less than ~ 400 ms Φ is indeed positively correlated with age as hypothesized (Fig 3). Φ is negative in this range, but for example, at 100 ms, the slope of Φ versus τ (Fig 2A) suggests that at ~ 51 weeks PMA, Φ would cross zero resulting in a positive contribution of causal holistic information after this age. τ also tended to be positively correlated with age for τ greater than ~ 1200 ms, where Φ was positive, though less significantly so. Interestingly where Φ was positive and maximal (τ between 700 and 800 ms), Φ was negatively correlated with age during sleep (Fig 3). This is discussed further below.

With regard to the effects of the Family Nurture Intervention (FNI) on Φ, of all effects found the FNI versus standard care group difference in developmental course has perhaps the most straightforward interpretation. FNI was conceived and developed within the Nurture Science Program at Columbia University to provide empirical evidence of effects first noted in clinical practice: namely, behaviors that facilitate reciprocal maternal/infant emotional connection improve outcome in babies born prematurely [19]. A randomized control trial of an intervention based on this theory (FNI) showed advanced brain maturation as indexed by 1) increased EEG power at term age [23], 2) decreased coherence in frontal regions [22], and 3) more rapid increases in EEG power from 35 to 40 weeks PMA [24]. Longer term outcomes from this study included improved 18-month cognitive and language scores, fewer emotional/behaviors problems, and reduced risk of autism [21]. In light of these results, we hypothesized that FNI would accelerate maturation of Φ. Indeed, slopes of Φ with age were steeper in FNI than control infants (Figs 4 and 5). In addition, except for very short values of τ (less than ~ 10 ms), we found larger and more significant state differences in Φ in the FNI group for both REM versus non-REM sleep (Fig 7) and wake versus sleep (Fig 8) suggesting FNI leads to better defined brain states. Together these findings provide further evidence that FNI accelerates brain maturation and that Φ is sensitive to these changes.

With regard to the overall effects of state on Φ, as hypothesized, values were lowest in quiet sleep and highest in waking, with values in active (REM) sleep intermediate for most values of τ (Figs 6, 7 and 8). These results were predominately at lower (< 10 ms) and higher (> ~ 800 ms) values of τ, with a cluster of significant effects for τ between 800 and 1200 ms. Interestingly, the sleep state dependency of Φ also occurred opposite to that hypothesized (i.e. Φ in QS > Φ in AS) in a fairly narrow intermediate range of τ. In contrast to these mixed sleep state dependencies, sleep versus wake comparisons were always as hypothesized when significant (Fig 8); however, the lower number of subjects with sufficient epochs of waking make these results less robust as there were fewer values of τ for which differences were significant.

**Fig 9:**
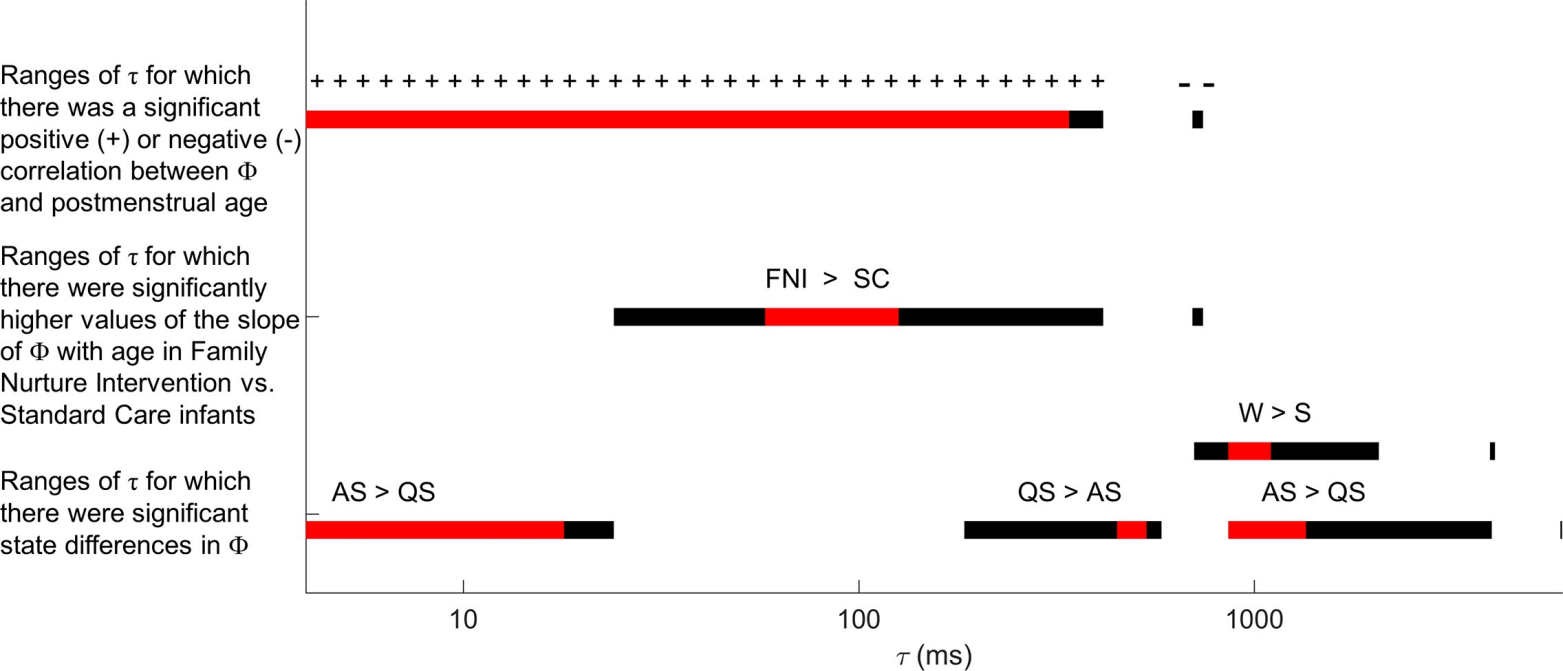
Visualization of ranges of temporal scale τ where significant effects were found. The top bar denotes correlations of Φ with postmenstrual age. The middle bar denotes FNI vs SC group differences in the slope of Φ with postmenstrual age. The bottom bars denote active (AS) vs quiet (AS) sleep and wake (W) versus sleep (S) state differences in Φ. In each case, effects with p-values < 0.05 are shown in black while effects with p-values < 0.0024 (Bonferroni correction) are shown in red.

To aid comparisons of the diverse findings, a conceptual summary is provided in Fig 9, highlighting the relationship between Φ and τ. The bar at the top shows values of τ for which Φ was positively correlated with post-menstrual age, supporting the developmental hypothesis (+’s above the bar), and indicates the range of τ for which this age-dependency of Φ was reversed, challenging the developmental hypothesis (-’s above the bar). The middle bar depicts the effects of the nurture intervention on the development of Φ, supporting the intervention hypothesis of accelerated brain maturation in the FNI group. The bottom bar depicts the dependence of wake and sleep states on Φ, indicating ranges of τ for which the state hypotheses were supported (Φ in W > Φ in QS > Φ in AS) or were not (Φ in QS > Φ in AS). Note the general continuity of the bars which reveal remarkable structure in effects’ dependence on lag, i.e. effects do not appear randomly related to τ. This justifies considering all effects with significance at p-values of 0.05 or less, shown in black. Nonetheless, bars are colored in red for those lags for which effects remained significant after Bonferroni correction for multiple comparisons, to highlight values of τ where effects were most robust. Considering only those lags, all results except one are consistent with our hypotheses (the sole exception being sleep state difference at τ = 500 ms).

Before discussing possible interpretations of these varied results, it is helpful to review what is known of the various temporal scales present in the human brain. Time series data can be modelled as autoregressive and/or moving average processes, as oscillations embedded in noise, or any of the three in combination. Evidence from EEG, MEG, and intracranial field potentials has shown that oscillations arising from locally (columnar-level) synchronized intracellular neuronal membrane potentials [31] underlie cognitive functions [32] and perhaps consciousness [33, 34]. Mechanisms whereby such oscillations enhance or suppress neuronal firing include: increased/decreased amplitudes, reflecting more or less gating of firing [35]; synchronization across brain regions, reflecting functional connectivity [36–38]; and phase-phase [39, 40] or phase-amplitude [41] cross-frequency coupling, reflecting hierarchical nesting of oscillations, perhaps due to hierarchical nesting of cognitive functions [42–44]. Although there is diversity in oscillatory mechanisms, they all contain an inherent time scale, namely the period of oscillation. Because τ is the lag at which Φ is computed, and Φ measures the causal holistic information within a dynamical network, it is reasonable to speculate that Φ at a particular τ is due to the fact that oscillations with that period increase predictability within a network. On the other hand, the power spectra of EEG, MEG and intracranial field potentials all exhibit “1/f” (power law) dependence on frequency, characteristic of scale free dynamics. From that perspective, τ may represent not the period of an oscillation, but rather the lag of an autoregressive process.

At the level of the neuron, the smallest temporal scales are those of ion channels in the cell membrane with time constants on the order of micro- or milliseconds. Temporal scales associated with chemical synapses between neurons are longer due to the times for neurotransmitter cleft traversal, receptor binding and dissociation, and reuptake. At higher levels of organization, networks of interconnected neurons have even larger temporal scales, such as the time for depolarization spikes to traverse the network, or the periods of a wide range of oscillations in the component cells’ membrane potentials noted previously.

From these considerations, one interpretation of the findings presented here is to reason that Φ at the lowest lags (<~ 20 ms) is related to macroscopically coordinated *intracellular* processes (ion channel and synaptic time constants) that can be modelled as autoregressive, non-oscillatory processes, while Φ at higher lags (>~ 350 ms) arise from *intercellular* oscillatory processes. Alternatively, another interpretation is that Φ at all values of τ is due to the information embedded in nested oscillatory processes and lower frequencies simply afford greater such embedding.

With these interpretations in mind, note that the increase of negative values of Φ with τ from 4 to 350 ms (Fig 1) roughly corresponds to the range of τ in which correlations of Φ with age are positive (Fig 3). Perhaps at higher inferred frequencies (i.e. lower τ), Φ reflects information embedded in non-oscillatory autoregressive processes while at lower frequencies (i.e. higher τ), Φ reflects the predictability inherent in functional oscillations. If true, one would predict a break in the “1/f” slope of power spectra at the frequency at which Φ transitioned from negative to positive, and that frequency would increase with age. We intend to explore this prediction in future work.

There were two discernable local maxima of Φ with τ (Fig 1) at ~ 720 and 3000 ms, corresponding to ~ 1.4 and 0.36 Hz. The perinatal EEG exhibits many stereotypical temporal patterns (trace alternans, delta brush, etc.) with large spectral content at/near those frequencies [45]. Perhaps peaks in Φ at those frequencies result from the predictability such patterns generate.

The finding that Φ in quiet sleep is higher than that in active sleep in the FNI cohort in the range of τ from ~200 to 600 ms (Fig 7A) was unexpected. Perhaps that is because the often-held view that infant active sleep is the analogue of adult REM sleep should be viewed with caution as is the opinion of other workers [46]. Adult REM is characterized by vivid dreams which are thought to consolidate episodic (narrative) memory of events during waking. In infants who spend 90% of their time asleep and have an unknown but perhaps weakly developed sense of self, active sleep may be relatively “dreamless”. If that were true, the emergence of dreams during active sleep should not occur until the percentage of time that infants spend awake reaches some threshold.

In this study, we investigated how well Φ correlates with states of consciousness as visually scored by trained state coders and found many values of τ with highly significant correlations. Although it may be argued that there already exist well known EEG correlates of conscious state (for example, increased high frequency power relative to low frequency power in REM sleep and waking compared to NREM sleep), conventional state determination typically uses more than one EEG measure together with behavioral measures such as eye movements and respiratory variability [47]. In fact, as far as we know, prior to this study, waking and REM sleep could not be distinguished using EEG alone. Furthermore, being a dynamical network measure defined over thousands of data samples that characterizes brain state with a single number, Φ represents an enormous data reduction strategy compared to conventional EEG measures that have values for every frequency and electrode lead and/or pair of leads, greatly simplifying post-hoc analyses.

Babies are a highly relevant population for studies of consciousness, both because they, like nonhuman species, are unable to provide verbal reports of their conscious state, and because they afford a means to investigate the ontogeny of consciousness. Here, a proposed measure of consciousness, integrated information or Φ, was applied to premature infant EEG data. Φ had a surprising degree of structure as a function of the lag over which it was computed. For most values of the lag, integrated information increased with age in early life and differed between conscious states, providing empirical support for IITC in human infants. Additionally, further evidence was found that facilitating emotional connection between mother and infants using FNI accelerates brain maturation in premature infants.
